# Significant Improvement of Fibromyalgia Symptoms With Osteopathic Manipulative Treatment: A Case Report

**DOI:** 10.7759/cureus.85471

**Published:** 2025-06-06

**Authors:** Ali Z Ansari, Nicholas A Mokodanski, Fizza Ahmed, Atqiya Fairooz, Sahar Hafeez

**Affiliations:** 1 Department of Pathology and Laboratory Medicine, William Carey University College of Osteopathic Medicine, Hattiesburg, USA; 2 Department of Family Medicine, Merit Health Wesley, Hattiesburg, USA; 3 Department of Family Medicine, Ross University School of Medicine, Bridgetown, BRB; 4 Department of Neurology, Lake Erie College of Osteopathic Medicine, Bradenton, USA; 5 Department of Family Medicine, University of South Florida, Tampa, USA

**Keywords:** chronic pain, complementary medicine, fibromyalgia, integrative medicine, musculoskeletal pain, nonpharmacologic therapy, osteopathic manipulative treatment, pain management, somatic dysfunction, symptom relief

## Abstract

Fibromyalgia is a chronic pain syndrome characterized by widespread musculoskeletal pain, fatigue, sleep disturbances, and cognitive dysfunction. While pharmacologic therapies and lifestyle modifications are commonly used, many patients experience suboptimal relief. We present the case of a 41-year-old African American female with a seven-year history of fibromyalgia, who sought care in a family medicine clinic for persistent pain, fatigue, and functional impairment despite ongoing pharmacologic management. Osteopathic manipulative treatment (OMT) was initiated during her visit, including soft tissue, myofascial release, articulatory, muscle energy, and high-velocity low-amplitude (HVLA) techniques applied to the cervical, thoracic, and lumbar spine, as well as the upper and lower extremities. The patient experienced immediate and significant relief following the first session, with direct OMT techniques seeming to have the most substantial impact in reducing symptoms. For six weekly sessions, all previously applied techniques were consistently repeated and adapted to address ongoing somatic dysfunction. The patient reported a reduction in pain intensity from 7/10 to 2/10, along with improvements in sleep quality, mood, and physical function. Post-treatment examination revealed decreased muscle tenderness and enhanced segmental mobility, with these improvements remaining stable at eight-week follow-up. This case highlights the potential of OMT, particularly direct techniques, to provide substantial symptom reduction in fibromyalgia patients, although lasting relief may require further interventions and follow-up.

## Introduction

Fibromyalgia is a chronic and often debilitating disorder characterized by widespread musculoskeletal pain, persistent fatigue, nonrestorative sleep, cognitive disturbances, and a range of somatic symptoms. It affects approximately 2%-4% of the population, with a higher prevalence among women [1]. Despite its high disease burden, the pathophysiology of fibromyalgia remains incompletely understood. Proposed mechanisms include central sensitization, in which the central nervous system becomes hypersensitive to pain stimuli, as well as dysfunction in the autonomic nervous system and the hypothalamic-pituitary-adrenal (HPA) axis [2]. Psychosocial factors such as emotional trauma, chronic stress, and mood disorders are also thought to play a contributory role in symptom onset and progression [3]. This multifactorial etiology presents a challenge to clinicians and often results in delayed diagnosis and fragmented care [4].

Management of fibromyalgia is inherently complex and typically involves a multidisciplinary approach. Standard treatment regimens include pharmacologic interventions such as serotonin-norepinephrine reuptake inhibitors (SNRIs), anticonvulsants like pregabalin, and tricyclic antidepressants [5]. Nonpharmacologic strategies, such as aerobic exercise, cognitive behavioral therapy, stress reduction techniques, and patient education, are equally important and are increasingly emphasized in clinical guidelines [6]. However, despite these therapeutic options, many patients continue to experience inadequate relief and report persistent pain and impaired quality of life [7]. This ongoing symptom burden has led to growing interest in integrative and complementary treatment modalities that can serve as adjuncts to conventional medical care [8].

Osteopathic manipulative treatment (OMT) represents one such approach. OMT is a hands-on therapeutic intervention employed by osteopathic physicians to diagnose, treat, and prevent somatic dysfunction through techniques aimed at improving structural alignment, reducing tissue tension, and enhancing physiologic function [9]. Among the variety of OMT modalities, direct techniques, including high-velocity low-amplitude (HVLA), muscle energy, and articulatory methods, are particularly noteworthy for their active engagement with the patient’s restrictive barriers. These methods involve placing the affected joint or tissue at its restrictive end-range and applying a therapeutic force, either by the physician (as in HVLA or articulatory techniques) or by the patient under guidance (as in muscle energy). These interventions are often used to address joint restrictions, reduce hypertonicity, and restore range of motion. When applied appropriately, direct OMT techniques may interrupt the cycle of somatic dysfunction and nociceptive feedback that contributes to chronic pain syndromes like fibromyalgia [10].

## Case presentation

A 41-year-old African American woman with a seven-year history of fibromyalgia presented to a family medicine clinic with persistent, widespread musculoskeletal pain and debilitating fatigue. Over the past three years, she reported a gradual yet marked worsening in both the frequency and intensity of her symptoms. She described the pain as “deep, dull, and constant,” primarily affecting her cervical, thoracic, and lumbar spine, as well as the bilateral shoulder girdles, hips, and pelvic region. On average, she rated her daily pain at 7/10 on the visual analog scale, with episodic exacerbations up to 8/10 - often triggered by physical exertion, emotional stress, changes in weather, or poor sleep.

In addition to chronic pain, the patient reported non-restorative sleep nearly every night, accompanied by morning stiffness lasting 60 to 90 minutes. She also experienced tension-type headaches two to three times per week, intermittent paresthesia in the upper extremities, and subjective cognitive dysfunction she described as “brain fog,” which included difficulties with memory, sustained attention, and word recall. To quantify symptom severity, we utilized elements of the Revised Fibromyalgia Impact Questionnaire (FIQR), which is a validated tool in assessing fibromyalgia. Her FIQR total was estimated at 68/100, reflecting significant functional impairment and symptom burden. Individual domain estimates included pain (7/10), fatigue (8/10), sleep quality (3/10), and cognitive clarity (4/10). Gastrointestinal symptoms consistent with irritable bowel syndrome (IBS) were also present, including alternating constipation and diarrhea, bloating, and abdominal cramping, with no identifiable dietary triggers. These cumulative symptoms severely impacted her quality of life and functional capacity, limiting her ability to maintain employment, complete household tasks, engage in physical activity, and care for her family. She denied recent trauma, fever, unintentional weight loss, or new neurologic deficits, but expressed growing emotional distress, frustration, and hopelessness due to the chronic and refractory nature of her condition.

Her past treatment history included multiple pharmacologic agents commonly used for fibromyalgia, including duloxetine (60 mg daily), amitriptyline (titrated from 10 to 25 mg nightly), gabapentin (up to 900 mg daily), and cyclobenzaprine (5 mg nightly as needed). These therapies offered only marginal relief and were ultimately discontinued due to adverse effects such as daytime sedation, dizziness, dry mouth, nausea, and bloating. Frustrated by the lack of sustained benefit and poor tolerability, she had also pursued several non-pharmacologic treatments. These included multiple courses of physical therapy targeting myofascial stretching, core strengthening, and posture retraining, though progress was often halted by pain exacerbations during and after sessions. She further explored integrative approaches such as acupuncture, guided meditation, mindfulness-based stress reduction, and dietary changes. While occasionally helpful in the short term, none yielded lasting or meaningful improvement. At the time of her presentation, she was no longer taking any prescription medications for fibromyalgia and used over-the-counter non-steroidal anti-inflammatory drugs (NSAIDs) such as ibuprofen on an as-needed basis. She denied alcohol, tobacco, or illicit drug use, had no history of opioid use or dependency, and no known family history of autoimmune, inflammatory, or connective tissue disorders.

On physical examination, she appeared alert, oriented, and cooperative. She ambulated independently but exhibited a mildly guarded gait, especially when transitioning from sitting to standing, suggestive of pain-related movement restriction. Postural evaluation was performed as part of the osteopathic structural exam, using standard clinical methods, including visual inspection from anterior, lateral, and posterior views, as well as palpation of bony landmarks and assessment during active motion. This revealed a subtle anterior head carriage, rounded shoulders, scapular protraction, and increased thoracic kyphosis with compensatory lumbar flattening. No scoliosis or pelvic obliquity was observed. Dynamic assessments revealed altered weight distribution and mild asymmetry in gait stride and pelvic rotation. Palpation of soft tissues uncovered diffuse myofascial tenderness, especially at classic fibromyalgia tender points-including the suboccipital muscles, upper trapezius, medial scapular borders, supraspinatus, second costochondral junctions, lateral epicondyles, gluteal muscles, greater trochanters, and medial knees. Hyperalgesia and mild allodynia were noted in several of these regions, assessed through light palpation and gentle stroking of the skin using the examiner’s fingertips. The patient reported discomfort or pain in response to stimuli that are normally non-painful, consistent with tactile allodynia. Additionally, she demonstrated paraspinal muscle hypertonicity and myofascial restriction, particularly in the cervical, upper thoracic, and lumbosacral areas, as determined through layer-by-layer palpation during the osteopathic structural exam. These findings were based on tactile assessment of increased muscle tone, decreased tissue compliance, and restricted fascial glide.

A focused neurologic exam was unremarkable. Cranial nerves II-XII were intact. Strength was preserved in all major muscle groups (5/5 bilaterally), with normal tone and bulk. Deep tendon reflexes were symmetric and normoactive (2+), and sensation to light touch, vibration, and pinprick was intact throughout. No cerebellar signs or coordination deficits were identified. Cardiopulmonary examination was normal, with regular S1 and S2 heart sounds and no murmurs, rubs, or gallops. Lung fields were clear bilaterally. The abdominal exam was soft, non-tender, and non-distended, with normal bowel sounds and no palpable masses or organomegaly. Musculoskeletal evaluation revealed no synovitis, joint deformities, or effusions.

Given the chronic, disabling nature of her symptoms and poor response to prior conventional therapies, the attending physician recommended a trial of OMT. The patient expressed enthusiasm for non-pharmacologic options and consented to proceed. A detailed osteopathic structural exam was performed, using layer-by-layer palpation and TART criteria (tissue texture changes, asymmetry, restriction of motion, and tenderness), revealing multiple significant somatic dysfunctions across both the axial and appendicular skeleton.

In the cervical spine, segments C2-C4 were flexed, rotated, and sidebent left (FRSL), with associated hypertonicity in the suboccipital and upper trapezial musculature. The thoracic spine from T3-T7 showed an extended, rotated, and sidebent right (ERSR) pattern with diminished rib cage motion on inspiration. The lumbar spine demonstrated a neutral group curve from T12-L3 with right rotation and left sidebending (NRRSL), along with tenderness and paraspinal texture changes. The pelvis exhibited a left anterior innominate rotation and a right superior shear, producing asymmetry. The sacrum demonstrated a right-on-right torsion (ROR) with restricted seated flexion. The rib cage revealed bilateral inhalation dysfunctions of ribs 2-6 and an exhalation dysfunction of rib 10 on the right. In the upper extremities, there was bilateral protraction of the shoulders and decreased range of motion with abduction and external rotation, indicating scapulothoracic and glenohumeral dysfunction. In the lower extremities, a posteriorly rotated left femoral head and bilateral piriformis hypertonicity with tenderness were noted. These findings collectively illustrated a pattern of global somatic dysfunction correlating with her reported pain and functional limitations (Table 1).

**Table 1:** The somatic dysfunctions identified during the patient’s initial osteopathic structural exam.

Region	Somatic dysfunction
Cervical spine	C2-C4 flexed, rotated, and sidebent left (FRSL); muscle hypertonicity in the suboccipital region and upper trapezius
Thoracic spine	T3-T7 extended, rotated, and sidebent right (ERSR); decreased rib cage motion on inspiration bilaterally
Lumbar spine	T12-L3 neutral, rotated right, sidebent left (NRRSL); palpable tenderness and paraspinal tissue texture changes
Pelvis	Left anterior innominate rotation; right superior shear dysfunction
Sacrum	Right-on-right torsion (ROR); restricted motion on seated flexion test
Ribs	Ribs 2-6 bilaterally inhaled (elevated); rib 10 exhaled on right
Upper extremities	Bilateral protracted shoulders; decreased range of motion with abduction and external rotation
Lower extremities	Left posteriorly rotated femoral head; bilateral piriformis hypertonicity and tenderness.

Treatment was initiated using a multimodal OMT approach, with an emphasis on direct techniques, including HVLA, muscle energy, and articulatory methods. These were chosen to address her most prominent somatic dysfunctions and restore biomechanical and neuromuscular balance. Supportive techniques such as soft tissue manipulation, myofascial release, and lymphatic drainage were also employed to promote tissue relaxation, improve circulation, and optimize the response to direct techniques.

Soft tissue techniques targeted the cervical, thoracic, and lumbar paraspinal muscles, as well as the gluteal and piriformis regions, reducing hypertonicity and improving motion. HVLA was applied to the T3-T5 and L2-L3 vertebrae and the sacroiliac joint, with audible cavitations and immediate subjective improvement. Muscle energy was used to address dysfunctions at C2-C4, the left anterior innominate, the ROR sacral torsion, and bilateral piriformis tension. Myofascial release techniques were applied at the thoracic inlet, suboccipital region, and diaphragm. Rib raising (T2-T7) and doming of the diaphragm were used to modulate autonomic tone and enhance lymphatic flow. Inhibition techniques (suboccipital release, sacral inhibition, and celiac ganglion release) were added to support parasympathetic activity. Articulatory techniques were used to restore motion in the glenohumeral joints and femoral heads. Each session concluded with thoracic and pedal lymphatic pump techniques to facilitate systemic fluid return. A summary of the OMT techniques applied is presented in Table 2.

**Table 2:** The direct and indirect OMT techniques applied to specific anatomical regions. OMT, osteopathic manipulative treatment

Technique	Region(s) treated
Soft Tissue	Cervical, thoracic, lumbar paraspinal musculature; gluteal and piriformis regions
High-Velocity Low-Amplitude (HVLA)	Thoracic and lumbar vertebrae (T3-T5, L2-L3); sacroiliac joint manipulation
Muscle Energy	Cervical (C2-C4), pelvic innominate rotation, sacral torsion, piriformis tension
Direct Myofascial Release	Thoracic inlet, suboccipital region, and diaphragm
Rib Raising	T2-T7 to balance sympathetic output
Doming of the Diaphragm	To enhance lymphatic return and improve respiratory excursion
Inhibition Techniques	Suboccipital release, sacral inhibition, celiac ganglion release
Articulatory Techniques	Glenohumeral joint and femoral head mobilization
Lymphatic Pump (Thoracic/Pedal)	To enhance lymphatic flow and reduce tissue congestion

The patient responded positively to the initial OMT session, reporting immediate relief of cervical and upper thoracic tension, which she described as a subjective sense of *lightness* and improved posture. These impressions were recorded through direct patient feedback immediately post-treatment and served as the foundation for ongoing symptom tracking. Encouraged by this outcome, she agreed to a structured six-week course of weekly OMT sessions. By the second session, she noted reduced morning stiffness, better sleep, and improved tolerance for daily activities. She also experienced clearer thinking and less fatigue. During the third week, she reported continued gains, including a decrease in pain to 5/10, better stamina, and fewer episodes of brain fog. By the fourth session, she described increased energy, improved posture, and enhanced ability to complete household chores with less fatigue. She especially credited techniques targeting spinal, pelvic, and rib dysfunctions for these improvements. Progress continued steadily through the series. By the final (sixth) session, her pain had decreased to 2/10, with sustained improvement in sleep, fatigue, mood, and cognitive clarity. She resumed light exercise and social activities. At eight weeks, these benefits persisted, and she transitioned to a biweekly OMT maintenance plan to support long-term symptom control. A summary of her symptom trajectory, based on serial 0-10 self-assessment scores for pain, fatigue, sleep quality, and cognitive clarity, is presented in Table 3.

**Table 3:** The patient’s subjective symptom scores across six weekly OMT sessions, based on a 0-10 scale (10 = most severe). Notable reductions in symptom severity were observed as early as week 2, with consistent improvement through week 6 and sustained benefit at the eight-week follow-up. *Sleep quality and cognitive clarity scores were based on patient self-assessment using a subjective 0-10 scale (10 = best). OMT, osteopathic manipulative treatment

Timepoint	Pain intensity (0-10)	Sleep quality (0-10)*	Fatigue (0-10)	Cognitive clarity (0-10)*
Initial presentation	7	3	8	4
After the first session	6	4	7	5
After the second session	5	6	6	6
After the third session	4	7	5	7
After the fourth session	3	7	4	7
After the sixth session	2	8	3	8
Eight-week follow-up	2	8	3	8

## Discussion

This case demonstrates a significant and sustained clinical response to OMT in a patient with longstanding fibromyalgia refractory to conventional pharmacologic and nonpharmacologic management. The patient presented with a complex array of symptoms - widespread pain, fatigue, nonrestorative sleep, cognitive dysfunction, and features consistent with IBS - which are consistent with the systemic, multi-symptom nature of fibromyalgia. Prior attempts at symptom control using serotonergic agents, tricyclic antidepressants, and antiepileptic medications were either ineffective or poorly tolerated, and adjunctive therapies such as physical therapy, acupuncture, and dietary modification produced only transient benefit. In this context, the patient’s rapid and progressive improvement following initiation of OMT is both clinically meaningful and mechanistically notable.

The therapeutic plan centered on a multimodal OMT approach, with particular emphasis on direct techniques, including HVLA, muscle energy, and articulatory methods, targeted to regions of documented somatic dysfunction. These techniques actively engaged the restrictive barrier, with therapeutic force applied either by the physician (HVLA, articulatory) or by the patient under guided resistance (muscle energy). In this case, direct techniques appeared to have the most immediate and reproducible clinical effect. The first session, which emphasized cervical and thoracic spine HVLA and muscle energy techniques for pelvic and sacral dysfunctions, was associated with immediate reductions in cervical and upper thoracic tension, improved posture, and decreased perceived pain intensity, as reported by the patient following treatment. While these initial improvements were based on subjective feedback, subsequent changes were quantified through serial 0-10 symptom ratings, which demonstrated consistent clinical progress over the six-week course. These outcomes suggest a rapid modulation of nociceptive input, likely through biomechanical realignment, restoration of joint motion, and attenuation of afferent sensitization at spinal and supraspinal levels [9,11].

Muscle energy techniques, in particular, were employed to address dysfunctional innominate rotation, sacral torsions, and hypertonic musculature in the cervical and pelvic regions. These techniques likely facilitated reciprocal inhibition, reduced muscular hypertonicity, and restored symmetry in joint and soft tissue motion-factors that may contribute to a downstream reduction in the central sensitization characteristic of fibromyalgia [12]. Similarly, HVLA techniques directed at thoracic and lumbar vertebrae may have improved segmental mobility and sympathetic regulation, especially given the thoracolumbar spine’s role in autonomic outflow. Dysregulation of the autonomic nervous system has been consistently implicated in the pathophysiology of fibromyalgia, with studies suggesting that heightened sympathetic tone and reduced parasympathetic activity contribute to the perpetuation of symptoms such as pain, sleep disturbances, and cognitive dysfunction [13]. The patient’s self-reported improvements in sleep quality, mood, and cognitive clarity following subsequent sessions may also be partially attributable to these effects on autonomic balance, consistent with prior literature suggesting that OMT can modulate sympathetic tone and enhance parasympathetic activity [10].

Complementary techniques such as myofascial release, soft tissue manipulation, rib raising, and lymphatic drainage were included to support the structural interventions and further promote homeostasis. These approaches likely contributed to a reduction in tissue congestion, improved lymphatic return, and normalization of regional circulatory dynamics-factors that are increasingly recognized in the holistic management of chronic pain syndromes [10]. Myofascial techniques, in particular, have demonstrated the ability to decrease interstitial pressure and facilitate drainage of inflammatory mediators, which may be elevated in fibromyalgia and contribute to widespread pain and tenderness [9]. However, it was the direct techniques that consistently coincided with the most pronounced symptom relief, particularly in terms of pain reduction and musculoskeletal function. This pattern supports the hypothesis that direct OMT modalities may play a central role in disrupting the mechanical and neurophysiological feedback loops that perpetuate fibromyalgia symptoms.

While this case provides valuable clinical insights, it must be interpreted with appropriate caution. The absence of a control group, the potential for placebo effect, and natural symptom variability inherent to fibromyalgia limit definitive conclusions regarding causality. Furthermore, the subjective nature of symptom reporting, though clinically important, poses challenges to standardization and reproducibility. Nonetheless, the consistency of the patient’s response across six weekly sessions, along with the sustained benefit observed at the eight-week follow-up, suggests more than a transient or coincidental effect.

This case adds to a growing body of anecdotal and early-stage evidence supporting the role of OMT, particularly direct manipulative techniques, as a viable and low-risk adjunctive therapy in fibromyalgia care. The hands-on, individualized nature of OMT aligns well with the principles of patient-centered medicine and may be especially valuable for patients with medication intolerance or those seeking alternatives to pharmacologic management. Moreover, given the complex interplay between musculoskeletal, autonomic, and central nervous system dysfunction in fibromyalgia, the multimodal impact of OMT offers a promising integrative therapeutic approach. Future controlled studies are warranted to further delineate the efficacy, optimal technique combinations, and physiologic mechanisms underlying OMT’s effects in fibromyalgia and other central sensitization syndromes.

## Conclusions

This case highlights the potential role of OMT as a valuable adjunct in the management of fibromyalgia, particularly for patients who have experienced limited benefit or intolerance to conventional therapies. The patient’s marked and sustained improvements in pain, fatigue, sleep quality, and cognitive function following a structured course of direct and supportive OMT techniques emphasize the therapeutic impact that hands-on, individualized care can provide. While these results cannot be generalized from a single case, they align with growing clinical interest in integrative, multimodal approaches for chronic pain syndromes. Given its low risk profile and potential for enhancing quality of life, OMT merits further investigation through controlled studies and may represent a viable addition to comprehensive fibromyalgia care when tailored to patient needs and somatic findings.

## References

[1] Wolfe F, Ross K, Anderson J, Russell IJ, Hebert L (1995). The prevalence and characteristics of fibromyalgia in the general population. Arthritis Rheum.

[2] Clauw DJ (2014). Fibromyalgia: a clinical review. JAMA.

[3] Thieme K, Turk DC, Flor H (2004). Comorbid depression and anxiety in fibromyalgia syndrome: relationship to somatic and psychosocial variables. Psychosom Med.

[4] Choy EH (2015). The role of sleep in pain and fibromyalgia. Nat Rev Rheumatol.

[5] Häuser W, Petzke F, Sommer C (2010). Comparative efficacy and harms of duloxetine, milnacipran, and pregabalin in fibromyalgia syndrome. J Pain.

[6] Macfarlane GJ, Kronisch C, Dean LE (2017). EULAR revised recommendations for the management of fibromyalgia. Ann Rheum Dis.

[7] Vincent A, Lahr BD, Wolfe F (2013). Prevalence of fibromyalgia: a population-based study in Olmsted County, Minnesota, utilizing the Rochester Epidemiology Project. Arthritis Care Res (Hoboken).

[8] Mist SD, Firestone KA, Jones KD (2013). Complementary and alternative exercise for fibromyalgia: a meta-analysis. J Pain Res.

[9] Licciardone JC, Gatchel RJ, Aryal S (2016). Recovery from chronic low back pain after osteopathic manipulative treatment: a randomized controlled trial. J Am Osteopath Assoc.

[10] Henley CE, Ivins D, Mills M, Wen FK, Benjamin BA (2008). Osteopathic manipulative treatment and its relationship to autonomic nervous system activity as demonstrated by heart rate variability: a repeated measures study. Osteopath Med Prim Care.

[11] Franke H, Franke JD, Fryer G (2014). Osteopathic manipulative treatment for nonspecific low back pain: a systematic review and meta-analysis. BMC Musculoskelet Disord.

[12] Licciardone JC, Schultz MJ, Amen B (2020). Osteopathic manipulation in the management of chronic pain: current perspectives. J Pain Res.

[13] Clauw DJ (2015). Fibromyalgia and related conditions. Mayo Clin Proc.

